# Managing the Cutaneous Sinus Tract of Dental Origine

**DOI:** 10.3889/oamjms.2016.100

**Published:** 2016-09-06

**Authors:** Edvard Janev, Enis Redzep

**Affiliations:** *Faculty of Dentistry, Ss Cyril and Methodius University of Skopje, Skopje, Republic of Macedonia*

**Keywords:** Fistula, cutaneous sinus tract, periapical dental infections, root canal

## Abstract

**BACKGROUND:**

Draining cutaneous sinus tract in chin area may be caused by chronic periapical dental infections. Misdiagnosis of these lesions usually leads to destructive invasive treatment of the sinus tract that is not correct and curative.

**CASE REPORT:**

A 31-year-old male patient referred to us with a chronically draining lesion on his chin. The lesion previously was misdiagnosed by medical doctors and had undergone two times surgery with a focus on the skin lesion and had received antibiotic therapy for a prolonged period of time. After clinical and radiologic examination the dental origin of the lesion was evident and proper endodontic and surgical treatment was performed. Three months later, after the treatment, the lesion showed total healing and reoccurrence occurred.

**CONCLUSION:**

The key to successful treatment of cutaneous sinus tract of dental origin must be in appropriate communication between the dentist and the physician in order to achieve correct diagnosis and therapy in such cases.

## Introduction

The successful treatment of cutaneous sinus tract of dental origin depends on the diagnosis of the source which may be very challenging. These lesions present a diagnostic problem and misdiagnosis leads to incorrect and unsuccessful treatment. Very often the possibility of an odontogenic origin is overlooked because most of the patients do not experience any dental symptoms.

Treatment with systemic antibiotics results in temporary cessation of the drainage which returns immediately after antibiotic treatment is over. The diagnosis may be challenging for several reasons:

1. The cutaneous lesions do not always arise in close proximity to the underlying infection and only half of all patients ever recall having had a toothache.
2. The sinus tract appears most commonly on the chin or jaw line but they also can appear elsewhere on the face and neck [1, 2].
3. Lesions have been reported to occur as far away from oral cavity as the chest, tight or sacrum [2-5].
4. Because cutaneous lesions can mimic other disorders, several inappropriate surgeries and courses of antibiotics are commonly used before definite therapy is instituted [2, 6, 7].

Patients with extraoral drainage from periapical pathosis may be unaware of any dental problem and tend to seek treatment from physicians, who may not give high priority to chronic dental infections.

The differential diagnosis should include:

- Infected pylar or epidermal cyst
- Carbuncle
- Pyogenic granuloma
- Suppurative lymphadenitis
- Foreign body reaction
- Thyroglossal tract fistula
- Branchial cleft fistula
- Actinomycosis
- Basal cell and squamous cell carcinoma [8-11]

Dental etiology of these lesions can be confirmed by:

- Tracing the sinus tract to its origin with gutta percha or other radioopaque material
- Pulp vitality testing
- Periapical films
- Panoramic films

## Case Report

A 31-year-old male patient referred to our clinic with a chronically draining lesion on his chin. His history revealed that he had this lesion for more than 5 months and had undergone two times surgery and received antibiotics for prolonged period of time.

**Figure 1:**
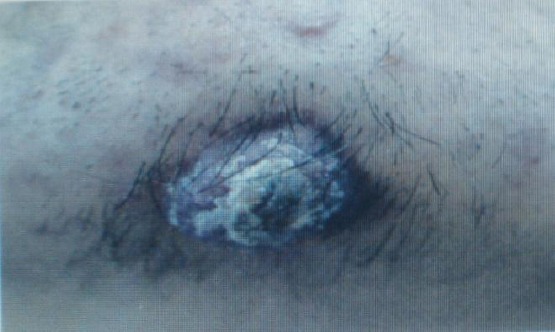
Skin lesion located on chin area

Dental history revealed no pain or any dental symptoms but he recalls to a direct blunt trauma to the anterior mandibular region. The periapical radiograph showed a large radiolucent area around lower right first incisor. There was no electric or thermal pulp testing performed on the same tooth. Neither percussion nor palpation revealed any abnormality.

**Figure 2:**
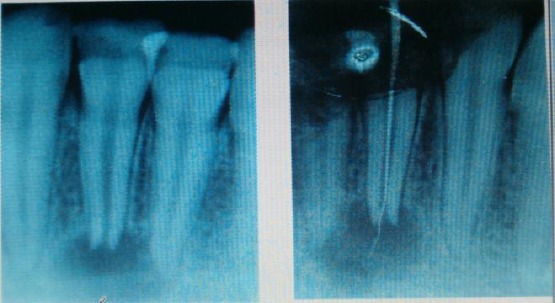
Rtg diagnosis of chronical periodontitis periapical and root canal treatment

The tooth was treated with calcium hydroxide and glycerine and antibiotics for 14 days. After the initial filling of the root canal, an apicoectomy and sinus excision was performed. Three months postoperative control revealed no sinus fistula or exudates from chin or from the mucosa.

**Figure 3:**
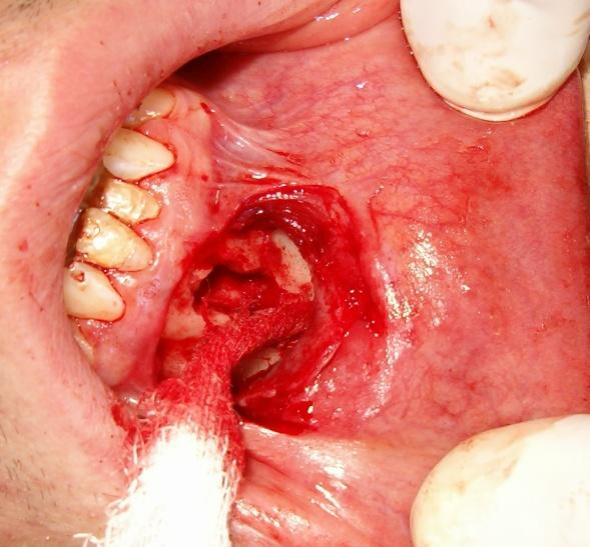
Surgical removal of periapical lesion with apical resection

## Discussion

If correctly diagnosed and treated the tract is expected to disappear within 7 to 14 days. Systemic antibiotic therapy will result in a temporary reduction of the drainage and apparent healing [12]. This tract however will recur immediately the AB therapy is completed unless the initial source is not eliminated [12].

**Figure 4:**
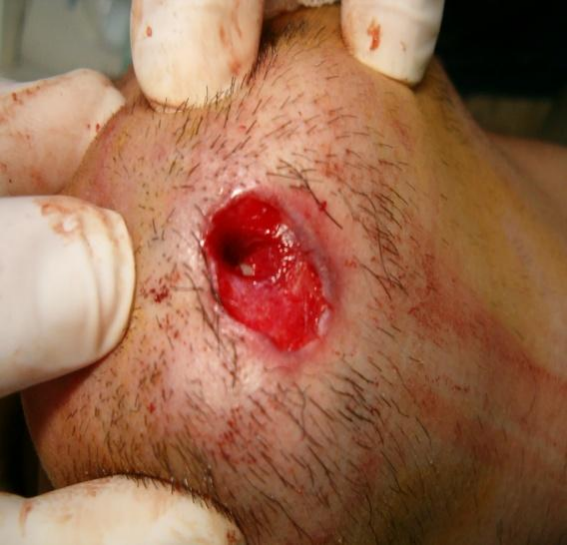
Curettage of the fistula

Extraoral cutaneous sinus tracts are usually lined with granulomatous tissue with a lumen containing a purulent exudate. The exudate is composed mainly of PMNL [13, 14]. Unlike intraoral tracts, extraoral tracts heal with granulation tissue leaving a cutaneous scar [15]. The patients may have to undergo a revision of the scar.

**Figure 5:**
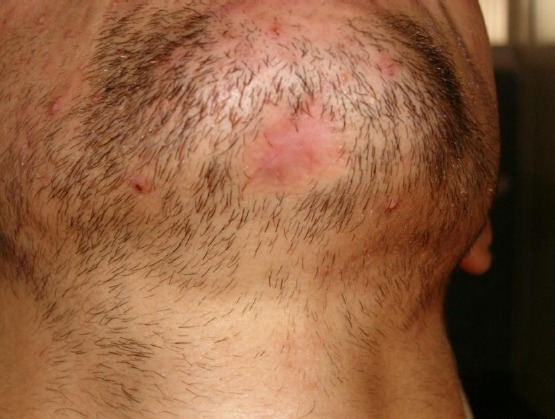
Total recovery in mental extraoral area a few weeks later

Eighty of reported cases of odontogenic origin are associated with mandibular teeth [16]. The sinus tract usually disappears in 5 to 14 days after the root canal system has been thoroughly cleansed [17]. An intraoral and extraoral sinus can develop depending on the path of the inflammation dictated by surrounding muscular attachments and facial planes [18].

The majority of sinuses arisen are intraoral [19, 20]. A retained root fragment can be the cause in edentulous patients [21, 22]. Most infections are polymicrobial and culture often yields growth of anaerobes or facultative anaerobes such as streptococcal spices [2, 19, 23, 24]. Due to two time’s surgical interventions and a prolonged antibiotic usage, we did not see relevant to have an antibiogram as it would not reflect the exact picture of the flora.

Johnson et al. believe that the application to heat to the face may contribute to the cutaneous exit of these sinus tracts since it is well known that the heat causes vasodilatation and increase blood flow to the local area [12]. Caliskan and colleagues presented three cases of cutaneous sinus tracts that were treated with CaOH and glycerine mixture intensionally placed beyond the apex. They performed microbiological culturing and found a mixed assortment of both obligate and facultative anaerobic bacteria identified as representatives of both endodontic abscesses and skin infections [25].

Conventional root canal therapy and sometimes extraction of the tooth are effective in achieving healing of cutaneous sinus tracts in a few weeks. In general, it is not necessary to treat the skin lesion, except for esthetic reason [26]. Al-Kandari reported completely healing of the sinus tract after proper root-canal treatment without surgical treatment in three months leaving a small scar [27].

In this case, the skin lesion was treated surgically because the patient had undergone two times surgical intervention with the focus on the skin lesion and had a bigger defect on his chin. Also, the time of healing is shortened with additional surgical removal and apical resection.

In conclusion, the key to successful treatment of cutaneous sinus tract of dental origin must be in appropriate communication between the dentist and the physician in order to achieve correct diagnosis and therapy in such cases. Basic principles of root canal treatment should be used judiciously to create a favourable environment while effectively eliminating the pathogens and giving the body’s immune, healing and repair mechanism a chance for the desired result.
